# A Functional Variant of PTPN22 Confers Risk for Vogt-Koyanagi-Harada Syndrome but Not for Ankylosing Spondylitis

**DOI:** 10.1371/journal.pone.0096943

**Published:** 2014-05-09

**Authors:** Qi Zhang, Jian Qi, Shengping Hou, Liping Du, Hongsong Yu, Qingfeng Cao, Yan Zhou, Dan Liao, Aize Kijlstra, Peizeng Yang

**Affiliations:** 1 The First Affiliated Hospital of Chongqing Medical University, Chongqing Key Laboratory of Ophthalmology and Chongqing Eye Institute, Chongqing, P. R. China; 2 University Eye Clinic Maastricht, Maastricht, The Netherlands; University of Birmingham, United Kingdom

## Abstract

**Background:**

Protein tyrosine phosphatase non-receptor 22 (*PTPN22*) is a key negative regulator of T lymphocytes and has emerged as an important candidate susceptibility factor for a number of immune-related diseases. This study aimed to examine the predisposition of PTPN22 SNPs to Vogt-Koyanagi-Harada (VKH) syndrome and acute anterior uveitis (AAU) associated with ankylosing spondylitis (AS).

**Methods:**

A total of 1005 VKH syndrome, 302 AAU^+^AS^+^ patients and 2010 normal controls among the Chinese Han population were enrolled in the study. Genotyping, PTPN22 expression, cell proliferation, cytokine production and cell activation were examined by PCR-RFLP, Real-time PCR, CCK8, ELISA and Flow cytometry.

**Results:**

The results showed significantly increased frequencies of the rs2488457 CC genotype and C allele but a decreased frequency of the GG genotype in VKH syndrome patients (P_Bonferroni correction_ (P_c_) = 3.47×10^−7^, OR = 1.54; P_c_ = 3.83×10^−8^, OR = 1.40; P_c_ = 6.35×10^−4^, OR = 0.62; respectively). No significant association of the tested SNPs with AAU^+^AS^+^ patients was observed. Functional studies showed a decreased PTPN22 expression, impaired cell proliferation and lower production of IL-10 in rs2488457 CC cases compared to GG cases (P_c_ = 0.009, P_c_ = 0.015 and P_c_ = 0.048 respectively). No significant association was observed concerning T cell activation and rs2488457 genotype.

**Conclusions:**

The study showed that a functional variant of PTPN22 confers risk for VKH syndrome but not for AAU^+^AS^+^ in a Chinese Han population, which may be due to a modulation of the PTPN22 expression, PBMC proliferation and IL-10 production.

## Introduction

Uveitis occurring in the context of systemic inflammatory diseases accounts for approximately half of the uveitis entities seen at a specialty clinic [1]. In Asia, the most three common systemic inflammatory diseases associated with uveitis are ankylosing spondylitis (AS), Vogt-Koyanagi-Harada (VKH) syndrome and Behcet's disease [2], [3], [4]. AS is known as a common inflammatory rheumatic disorder associated with characteristic inflammatory back pain, enthesitis, asymmetrical peripheral oligoarthritis, and specific organ attacks related to acute anterior uveitis (AAU), psoriasis and chronic inflammatory bowel disease [5]. VKH syndrome, a systemic granulomatous inflammatory illness, usually manifests as bilateral panuveitis associated with extraocular findings involved in tegumentary, hairy, auditory, and central nervous system signs [6]. Behcet's disease, a multisystem inflammatory disease, is usually characterized by recurrent uveitis, oral ulceration, arthritis, genital ulceration, skin lesions, and vascular inflammation [7]. The three are usually considered to be immune-related diseases. The pathogenesis of these disorders are yet indistinct, but genetic predisposition, environmental factors and the innate immune system are presumed to be interactively involved in their complex pathogenesis [8], [9], [10], [11], [12]. Human leukocyte antigen (*HLA*) genes, such as *HLA*-*B27, HLA*-*DR4* and *HLA*-*B51*, have been shown to be genetic predisposing factors for certain uveitis entities. However their contribution to the genetic risk is still limited and does not fully explain the genetic association. This has been the reason for a further analysis of non-*HLA* genes, with an emphasis on genes involved in the immunological and inflammatory response.

The protein tyrosine phosphatase non-receptor 22 (*PTPN22*) gene encodes the lymphoid-specific phosphatase known as Lyp, which contains a non-catalytic C-terminus composed of four proline-rich domains and a catalytic N-terminal domain. By interacting with Csk (C-terminal Src kinase), ZAP70 (zeta-associated protein-70) and Vav (a guanine-nucleotide exchange factor for the GTPases) involved in the TCR (T-cell receptor) signaling pathway, Lyp plays an important suppressive role of T cell responses [13], [14], [15], [16], [17]. *PTPN22* has been shown to be one of the strongest non-HLA susceptibility genes for various autoimmune diseases, such as rheumatoid arthritis (RA), type 1diabetes (T1D), systemic lupus erythematosus (SLE), and Graves' disease (GD) [18], [19], [20], [21], [22]. In several earlier studies, the main point of these association studies is the *PTPN22* SNP +1858C/T (rs2476601). The *PTPN22* 1858C/T polymorphism shows a large variation among ethnic groups and appears to be low in Asian and African populations and is virtually absent in Chinese Han [23], [24], [25], [26]. In these latter populations it is possible that SNPs that are in linkage disequilibrium with the 1858C/T SNP (rs2476601) or even other functional variants of *PTPN22* might be involved in the pathogenesis of autoimmune disease [27], [28]. In Asian populations, SNPs named rs2488457, rs3789604 and rs1310182, have been associated with several immune diseases, such as T1D, GD, RA and primary immune thrombocytopenia (ITP) [21], [29], [30], [31].

We recently evaluated the contribution of *PTPN22* gene polymorphisms (rs2488457, rs1310182 and rs3789604) to ocular Behcet's disease in Chinese and were not able to detect a significant association [32]. We now extend these studies on *PTPN22* gene polymorphisms in two other frequently observed uveitis entities in China, namely VKH syndrome and anterior uveitis associated with ankylosing spondylitis. For this study we chose three SNPs (rs2488457, rs1310182 and rs3789604) based on earlier literature and allele frequency in the Chinese Han population and showed that rs2488457 (-1123G/C) confers a significant risk for VKH syndrome. Functional analysis of this allele suggested that the polymorphism of SNP rs2488457 may involved in the development of VKH disease.

## Materials and Methods

### Study subjects

A total of 302 AAU^+^AS^+^ patients and 1005 VKH syndrome patients of Chinese Han ethnicity, were gathered from the Uveitis Study Center of the Sun Yat-sen University (Guangzhou, China) and the First Affiliated Hospital of Chongqing Medical University (Chongqing, China) between January 2005 and February 2013. The diagnoses of AS and VKH syndrome were strictly according to the Modified New York Criteria 1984 for AS and the Revised diagnostic criteria 2001 for VKH syndrome [33], [34]. We included 2010 healthy individuals, matched with the patients in gender, age, race and geographical origin. Genotype frequencies of the tested SNPs in the controls are complied with the Hardy-Weinberg equilibrium (HWE).

### Ethics statement

The study protocol was approved by the Ethics Committee of the First Affiliated Hospital of Chongqing Medical University, Chongqing, China (Permit Number: 2009-201008), and all processes were in agreement with the Declaration of Helsinki. Blood samples could not be collected until informed consent was signed by each participant.

### Genomic DNA extraction and genotyping

All peripheral blood samples were gathered in EDTA tubes and stored at −70°C. The methods for DNA extraction and genotyping were described in our previous study [32]. Direct sequencing was performed for approximately randomly selected 5% of samples to confirm the validity of the genotyping method used.

### Cells isolation and culture

Peripheral blood mononuclear cells (PBMCs) were separated by Ficoll-Hypaque density gradient centrifugation. The PBMCs were resuspended at a concentration of 1×10^6^ cells/ml and treated with LPS (100 ng/ml, Sigma, Missouri, USA) for 24 h to stimulate TNF-α, IL-1β, IL-6, IL-8 and MCP-1 production. For stimulation of IFN-γ, IL-10 and IL-17 production, the PBMCs were treated with anti-CD3 (OKT3, 0.5 µg/ml) and anti-CD28 antibodies (15E8, 0.1 µg/ml) (Miltenyi Biotec, Palo Alto, CA) for 72 h.

### RNA preparation and real-time quantitative PCR

Total RNA was extracted from PBMCs with TRIzol (Invitrogen, Carlsbad, CA) according to the manufacturer's instructions. Real-time PCR was performed on the ABI7500 Fast System (Applied Biosystems). The primers used for PTPN22 and β-actin detection have been described elsewhere [32], [35]. The relative expression of PTPN22 was normalized to the expression of the internal control β-actin using the 2^−ΔΔCT^ method.

### Cell proliferation assay

PBMCs stimulated with anti-CD3/CD28 antibodies (5∶1) (Miltenyi Biotec, Palo Alto, CA) were incubated for 72 h. Cell proliferation was examined with Cell Counting Kit-8 (CCK8) (Sigma-Aldrich, St Louis, MO) according to the manufacturer's instructions. The absorbance was determined at 450 nm using a Microplate Reader (SpectraMax M2e, Molecular Devices, USA).

### Measurement of cytokines

Supernatants of the stimulated PBMCs were collected for cytokine detection. The production of TNF-α, IL-1β, IL-6, IL-8, MCP-1, IFN-γ, IL-10 and IL-17 was measured with Duoset ELISA development kits (R&D Systems, Minneapolis, MN) according to the manufacturer instructions.

### Flow cytometry analysis

In order to determine the activation of CD4^+^ T cells, PBMCs were incubated with FITC-conjugated anti-human CD4, APC-conjugated anti-human CD44, PE-conjugated anti-human CD25, PE-cy7-conjugated anti-human CD69 or appropriate isotypes (eBioscience, San Diego, CA) for 30 minutes at 4°C. FACScan flow cytometer (BD Biosciences, San Diego, CA) and FlowJo software (Tree Star, Inc. Ashland, USA) were used for flow cytometry analysis.

### Statistical analysis

Genotype frequencies were calculated by direct counting. HWE was examined with the chi-square test. Allele and genotype frequencies in patients and controls were compared by the chi-square test with SPSS (v. 17.0; SPSS Inc., Chicago, IL). The P values were corrected (Pc) with the Bonferroni correction by multiplying with the number of comparisons performed. The results of gene expression, cytokine expression, cell proliferation and the activation of CD4+T cells were analyzed by Student's t test or Nonparametric Mann-Whitney U-test. Values were considered to be significantly different when P<0.05. Data are expressed as mean ± SD or mean ± SEM.

## Results

### Clinical feature of AAU^+^AS^+^ and VKH syndrome patients

The demographic characteristics and clinical features of the enrolled VKH syndrome and AAU^+^AS^+^ patients are shown in Table S1-S3 (Supporting Tables).

### Genotype and allele frequencies of *PTPN22* polymorphisms in patients and controls

Three SNPs of *PTPN22* (rs2488457, rs1310182 and rs3789604) were genotyped in 302 AAU^+^AS^+^ patients, 1005 VKH syndrome patients, and 2010 healthy controls. A significantly increased frequency of the CC genotype and C allele of rs2488457, and a decreased frequency of the GG genotype in VKH syndrome patients (P_c_ = 3.47×10^−7^, OR = 1.54; P_c_ = 3.83×10^−8^, OR = 1.40; P_c_ = 6.35×10^−4^, OR = 0.62; respectively) (Table 1) were identified. No significant association was found for the other two SNPs and VKH syndrome patients (Table 1). Similarly, we failed to find a significant association of the three *PTPN22* SNPs with AAU^+^AS^+^ (Table S4).

**Table 1:** Effects of PTPN22 SNPs on VKH syndrome risk.

Genotype	VKH		Controls		P value	Pc value	OR (95%CI)
	N = 1005		N = 2010				
rs2488457	N	%	N	%			
GG	102	10.1	310	15.4	7.06×10^−5^	6.35×10^−4^	0.62(0.49–0.79)
CG	454	45.2	1009	51.2	0.009	NS	0.82(0.70–0.95)
CC	449	44.7	691	34.4	3.86×10^−8^	3.47×10^−7^	1.54(1.32–1.80)
G Allele	658	32.7	1629	40.5	4.25×10^−9^	3.83×10^−8^	0.71(0.64–0.80)
C Allele	1352	67.3	2391	59.5	4.25×10^−9^	3.83×10^−8^	1.40(1.25–1.57)
rs1310182							
CC	48	4.8	84	4.2	0.450	NS	1.15(0.80–1.65)
CT	292	29	589	29.3	0.887	NS	0.99(0.84–1.17)
TT	665	66.2	1337	66.5	0.849	NS	0.99(0.84–1.16)
C Allele	388	19.3	757	18.8	0.659	NS	1.03(0.90–1.18)
T Allele	1622	80.7	3263	81.2	0.659	NS	0.97(0.85–1.11)
rs3789604							
TT	648	64.5	1254	62.4	0.262	NS	1.09(0.94–1.28)
GT	303	30.1	651	32.4	0.231	NS	0.90(0.77–1.06)
GG	54	5.4	105	5.2	0.863	NS	1.03(0.74–1.44)
T Allele	1599	79.6	3159	78.6	0.384	NS	1.06(0.96–1.21)
G Allele	411	20.4	861	21.4	0.384	NS	0.94(0.83–1.08)

P_c_ = Bonferroni corrected P value. NS = Not significant. OR = odds ratio. 95% CI = 95% confidence interval.

### Linkage disequilibrium (LD) data of the SNPs used

The LD data for the three SNPs (rs2488457, rs1310182 and rs3789604) investigated in this study showed no linkage disequilibrium. Values of the pair-wise D' and *r* ^2^ are shown in blocks (Figure S1).

### The influence of rs2488457 on the expression of *PTPN22*

**Figure 1:**
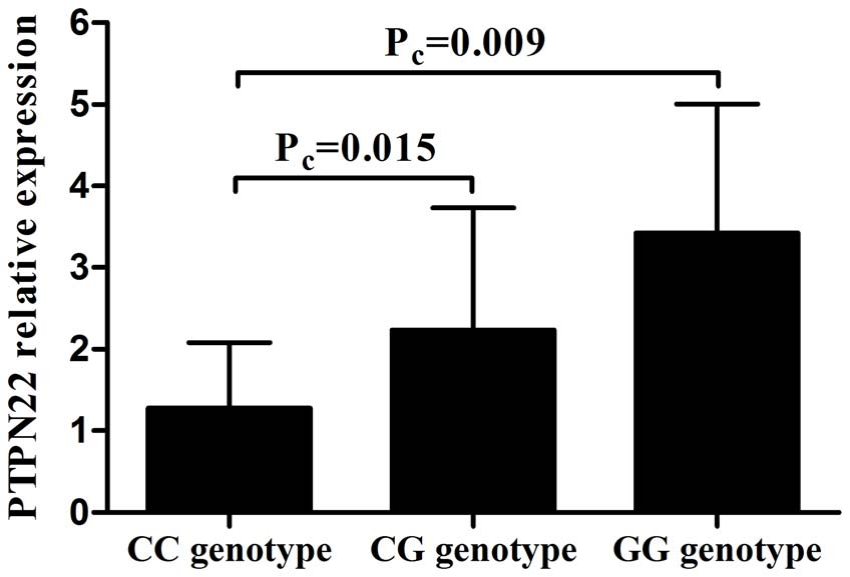
The influence of rs2488457 on the mRNA expression of PTPN22. The mRNA expression of PTPN22 in PBMCs from normal controls carrying different genotypes of rs2488457 (CC = 18, CG = 31, GG = 9). Data are represented as the mean ± SD.

To investigate whether the expression of PTPN22 was affected by the different genotypes of rs2488457 we performed the following experiments. PBMCs were isolated for *PTPN22* detection from 58 unrelated genotyped healthy individuals (CC = 18, CG = 31, GG = 9). Our results showed a significantly decreased expression of PTPN22 in CC cases compared to CG and GG cases (Figure 1. P_c_ = 0.015; P_c_ = 0.009, respectively).

### The influence of rs2488457 on the proliferation of PBMCs

**Figure 2:**
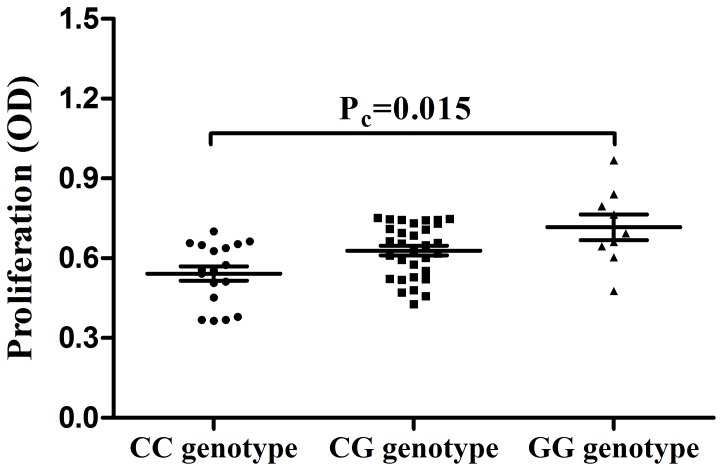
The influence of rs2488457 on the proliferation of PBMCs. The proliferation of anti-CD3/CD28 stimulated PBMCs from normal controls carrying different genotypes of rs2488457 (CC = 18, CG = 31, GG = 9). Data are represented as the mean ± SEM.

Since the role of *PTPN22* on immune cell proliferation is still controversial, we examined the influence of rs2488457 on the proliferation of PBMCs. PBMCs used for proliferation experiments were obtained from 58 unrelated genotyped healthy individuals (CC = 18, CG = 31, GG = 9). The results showed a significantly decreased proliferation in CC cases compared to GG cases following in vitro stimulation with anti-CD3/CD28 antibodies (Figure 2. P_c_ = 0.015).

### The influence of rs2488457 on the cytokine production

**Figure 3:**
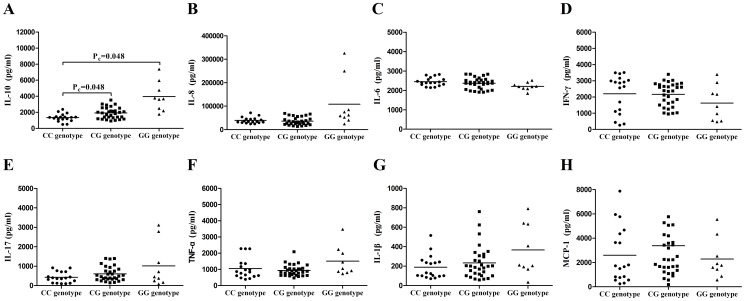
The influence of rs2488457 on the cytokine production. The production of IL-10(A), IL-8(B), IL-6(C), IFN-γ(D), IL-17(E), TNF-α(F), IL-1β(G) and MCP-1(H) by PBMCs from normal controls carrying different genotypes of rs2488457 (CC = 18, CG = 31, GG = 9). Data are represented as the mean.

Cytokines play a critical role in the pathogenesis of uveitis and we therefore investigated whether the different genotypes of rs2488457 affected production cytokines such as TNF-α, IL-1β, IL-6, IL-8, MCP-1, IFN-γ, IL-10 and IL-17. PBMCs were obtained from 58 unrelated genotyped healthy individuals (CC = 18, CG = 31, GG = 9) and stimulated with LPS or anti-CD3/CD28 antibodies, then supernatants were collected for cytokine analysis. A significantly decreased production of IL-10 by stimulated PBMCs was observed in CC cases compared to CG or GG cases (Figure 3. A P_c_ = 0.048; P_c_ = 0.048, respectively). Although a decreased IL-8 and an increased IL-6 production by stimulated PBMCs were observed in CC cases compared to GG cases (Figure 3. B–C), significance was lost after correction for multiple comparisons. No significant association was observed concerning IFN-γ, IL-17, TNF-α, IL-1β and MCP-1 production by stimulated PBMCs with the different genotypes of rs2488457 (Figure 3. D–H).

### The influence of rs2488457 on the activation of CD4^+^ T cells

**Figure 4:**
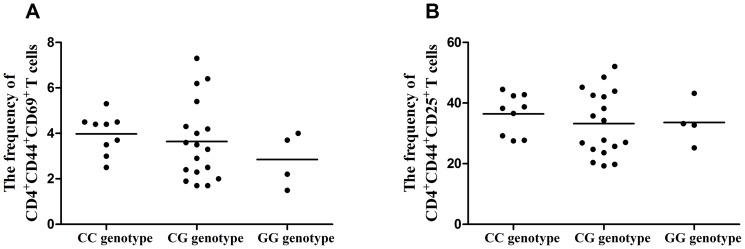
The influence of rs2488457 on the activation of CD4+ T cells. (A) The frequency of CD4^+^CD44^+^CD69^+^ T cells from normal controls carrying different genotypes of rs2488457 (CC = 9, CG = 18, GG = 4). (B) The frequency of CD4^+^CD44^+^CD25^+^ T cells from normal controls carrying different genotypes of rs2488457 (CC = 9, CG = 18, GG = 4). Data are represented as the mean.

Previous studies showed that *PTPN22* knockout mice accumulate activated T cells. To investigate the role of rs2488457 on T cell activation, we examined the early and late activation markers of CD4^+^ T cells in carriers of the different genotypes of rs2488457. PBMCs used for the detection of T cell activation were obtained from 31 unrelated genotyped healthy individuals (CC = 9, CG = 18, GG = 4). No significant association was observed concerning the frequencies of CD4^+^CD44^+^CD69^+^ and CD4^+^CD44^+^CD25^+^ T cells in the different genotypes of rs2488457 (Figure 4).

## Discussion

In the present study, we show that a functional variant rs2488457 of *PTPN22* is associated with a higher risk for the development of VKH syndrome. A functional rationale is provided since the risk genotype modulates *PTPN22* expression, PBMC proliferation and IL-10 production. Our study confirms earlier findings whereby *PTPN22* has emerged as a critical candidate susceptibility gene for amount of immune-related diseases [36], [37], [38]. Most studies on the association between immune disorders and *PTPN22* showed an association with the 1858C/T rs2476601 polymorphism [37], [39]. This polymorphism is absent in Chinese Han, but the rs2488457 (-1123G/C) has been shown to be in linkage disequilibrium (LD) with rs2476601 and this area of the gene may have functional consequences [25], [28]. Further studies are needed to clarify the exact molecular mechanisms involved.

We showed that rs2488457 had an effect on the *PTPN22* expression, PBMCs proliferation and cytokine production. We only investigated the effect of rs2488457 in healthy controls since the patients do not represent a homogenous sample due to a variable disease course and the fact that they are often treated with immunosuppressive agents. We observed a decreased expression of *PTPN22* in individuals carrying the rs2488457 CC genotype. Although the exact mechanism whereby rs2488457 modulates disease susceptibility remains unknown, our results indicate that this SNP may change the transcriptional activity of the *PTPN22* gene. Of interest is the fact that the DNA sequence around rs2488457 exactly matches with the binding site for the transcription factor activator protein 4 (AP-4) [27]. Further studies are needed to examine whether AP-4 affects the transcription activities of *PTPN22. PTPN22* is critically involved in the TCR signaling pathway [13], [16] and proliferation is one of consequences of TCR signaling. We therefore studied whether rs2488457 could affect the proliferation of PBMCs. The results showed that a decreased proliferation of PBMCs in rs2488457 CC cases as compared to GG cases when the cells were stimulated in vitro with a combination of anti-CD3/CD28 antibodies. Others have shown a decreased proliferation of CD4^+^T cell in 1858C/T which would be in agreement with our findings when considering that this locus is in linkage disequilibrium with rs2488457 [40]. Our results also revealed that the production of IL-10 from individuals carrying the rs2488457 CC genotype was significantly decreased compared to CG and GG carriers. A similar association of the 1858C/T polymorphism with a decreased IL-10 production has been demonstrated in a previous study [41]. The report showed that IL-10 gene knockout mice develop autoimmune disease which indicated that IL-10 played a critical role in autoimmunity [42]. The decreased production of IL-10 from individuals carrying the rs2488457 CC genotype fits in with the predisposing role of this genotype for VKH syndrome. Although the important role of *PTPN22* in the activation of T cells has been reported in a transgenic animal model [43], our results failed to find an association of rs2488457 with the activation of T cells. One possible reason for this discrepancy could be due to species differences (human versus mouse).

AS, VKH syndrome and Behcet's disease are three common systemic inflammatory diseases associated with uveitis seen in Asia [2], [3], [4]. Very recently, our group revealed that *PTPN22* gene polymorphism did not confer risk for ocular Behcet's disease [32], but the role of *PTPN22* gene polymorphisms in other uveitis entities such as AAU^+^AS^+^ and VKH syndrome in Chinese Han was not yet clear.

For several autoimmune diseases characterized by specific autoantibodies like rheumatoid arthritis, type 1 diabetes and systemic lupus erythematosus, a significant association with PTPN22 gene polymorphisms has been demonstrated by several groups [18], [19], [20]. However, a number of other immune diseases without specific autoantibodies did not show an association with *PTPN22* gene polymorphisms, such as multiple sclerosis, ulcerative colitis, Crohn's disease and systemic sclerosis [44], [45], [46], [47]. *PTPN22* +1858C/T SNP was also not associated with AS in a Spanish population nor with AAU^+^AS^+^ in American patients [48], [49]. On the other hand a Taiwanese study recently showed that the PTPN22 CC and GC genotypes of rs2488457 had a higher risk of AS than individuals with the GG genotype [relative risk  = 1.39, 95% confidence interval (95% CI) 1.03–1.88) [50]. We were not able to find an association with PTPN22 gene polymorphisms (including rs2488457) and AAU+AS+. The reason for the discrepancy with the Taiwanese study is not clear but may be caused by the fact that we only included AS patients with uveitis. Furthermore the Taiwanese patients originated China's South-East coastal areas, while our cases mainly came from China's Midwest areas. Six other SNPs (rs3811021, rs1217413, rs1237682, rs3761935, rs3789608, and rs2243471) were reported not associated with VKH disease in Japan [51]. These results did not include the associated SNP rs2488457 found in our study. The different conclusions between the Japanese study and ours might be due to differences in ethnicity or due to differences in sample size.

As mentioned above, our study showed a strong association of *PTPN22*/rs2488457 with VKH syndrome but not with AAU^+^AS^+^. Why certain uveitis entities are associated with PTPN22 gene polymorphisms and others are not remains unclear. AS is currently classified as an autoinflammatory disease, whereas VKH syndrome is considered as an autoimmune disease directed against melanocytes [11], [52]. In an earlier study, we failed to find an association between *PTPN22* polymorphisms and ocular Behcet' disease[32]. Behcet' disease is also thought to be an autoinflammatory disease. The lack of an association between *PTPN22* polymorphisms with both AAU^+^AS^+^ and ocular Behcet's disease suggests that the PTPN22 association with uveitis may be confined to those entities that involve an autoimmune pathogenesis.

There are also several limitations to consider in this study. First of all, the enrolled patients and controls all belong to a Chinese Han population. Further multi-ethnic and multicenter studies should be performed in the future to confirm our data. Secondly, the enrolled patients in this study were all recruited from our uveitis clinic. In view of the multiple organ involvement in VHK syndrome and AS, further studies are needed to examine the association of *PTPN22*/rs2488457 with VKH syndrome and AS patients recruited from other medical departments. It would be interesting to study the functional effect of the other SNPs used in our study. Since we only found an association of SNP rs2488457 with VKH disease and since there was no linkage disequilibrium among the three SNPs used in our study (rs2488457, rs1310182 and rs3789604), we confined the functional analysis to rs2488457.

In conclusion, our results show that a functional variant rs2488457 of the *PTPN22* gene is associated with an increased risk for the development of VKH syndrome by modulating the gene expression, PBMC proliferation and IL-10 production.

## Supporting Information

Figure S1 **Pair-wise linkage disequilibrium values of PTPN22 SNPs in a Chinese Han population.** (A) Values of the pair-wise D' (×100) are shown in blocks. (B) Values of the pair-wise *r* ^2^ (×100) are shown in blocks.
(TIF)

Table S1 **Characteristics of the investigated healthy controls.**
(DOC)

Table S2 **Clinical features of the VKH syndrome patients.**
(DOC)

Table S3 **Clinical features of the AS patients.**
(DOC)

Table S4 **Effects of PTPN22 SNPs on AAU^+^AS^+^ risk.**
(DOC)
