# Supplementary figures and images for: A Functional Variant of PTPN22 Confers Risk for Vogt-Koyanagi-Harada Syndrome but Not for Ankylosing Spondylitis
Source: PLoS One. 2014 May 9;9(5):e96943. doi: 10.1371/journal.pone.0096943 (PMC4016172; doi:10.1371/journal.pone.0096943)

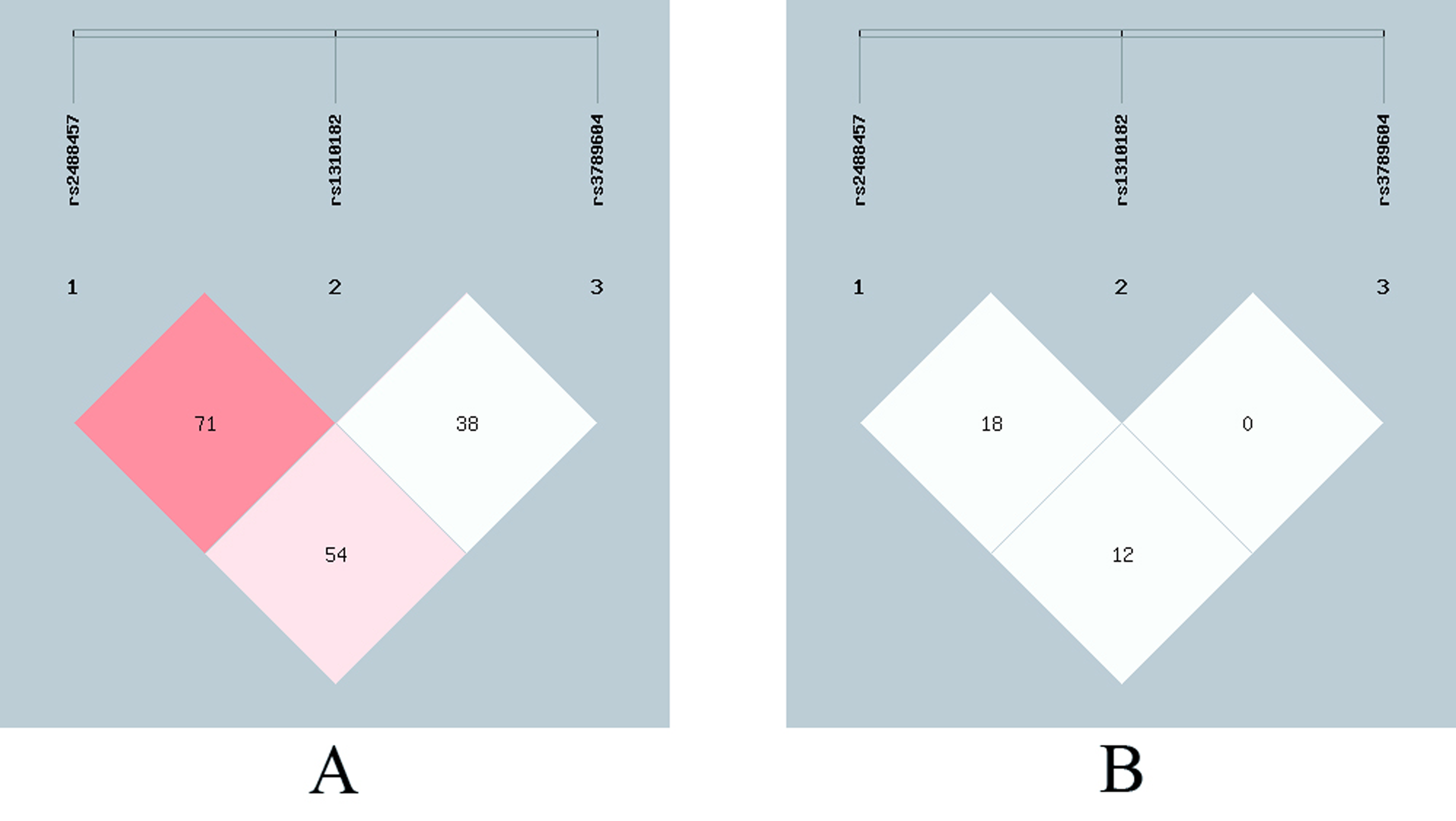

Supplement: Figure S1 — Pair-wise linkage disequilibrium values of PTPN22 SNPs in a Chinese Han population. (A) Values of the pair-wise D' (×100) are shown in blocks. (B) Values of the pair-wise r 2 (×100) are shown in blocks. (TIF) [file pone.0096943.s001.tif]
