# Supplementary material for: A Functional Variant of PTPN22 Confers Risk for Vogt-Koyanagi-Harada Syndrome but Not for Ankylosing Spondylitis
Source: PLoS One. 2014 May 9;9(5):e96943. doi: 10.1371/journal.pone.0096943 (PMC4016172; doi:10.1371/journal.pone.0096943)
Supplement: Table S1 — Characteristics of the investigated healthy controls. (DOC) [file pone.0096943.s002.doc]

**Table S1. Characteristics of the investigated healthy controls**

| **Characteristics** | **Healthy Controls** | |
| --- | --- | --- |
|  | N(total=2010) | % |
| Age (years±SD) | 37.26±10.58 |  |
| Male | 1135 | 56.5 |
| Female | 875 | 43.5 |
