# Supplementary material for: A Functional Variant of PTPN22 Confers Risk for Vogt-Koyanagi-Harada Syndrome but Not for Ankylosing Spondylitis
Source: PLoS One. 2014 May 9;9(5):e96943. doi: 10.1371/journal.pone.0096943 (PMC4016172; doi:10.1371/journal.pone.0096943)
Supplement: Table S2 — Clinical features of the VKH syndrome patients. (DOC) [file pone.0096943.s003.doc]

**Table S2. Clinical features of the VKH syndrome patients**

| **Clinical features** | **Patients with VKH syndrome** | |
| --- | --- | --- |
|  | N(total=1005) | % |
| Age (years±SD) | 35.68±12.76 |  |
| Male | 553 | 55.0 |
| Female | 452 | 45.0 |
| Uveitis | 1005 | 100 |
| nuchal rigidity | 148 | 14.7 |
| headache | 431 | 42.9 |
| scalp allergy | 183 | 18.2 |
| tinnitus | 501 | 49.9 |
| hearing loss | 362 | 36.0 |
| alopecia | 456 | 45.4 |
| gray hair | 423 | 42.1 |
| vitiligo | 212 | 21.1 |
