# Supplementary material for: A Functional Variant of PTPN22 Confers Risk for Vogt-Koyanagi-Harada Syndrome but Not for Ankylosing Spondylitis
Source: PLoS One. 2014 May 9;9(5):e96943. doi: 10.1371/journal.pone.0096943 (PMC4016172; doi:10.1371/journal.pone.0096943)
Supplement: Table S3 — Clinical features of the AS patients. (DOC) [file pone.0096943.s004.doc]

**Table S3. Clinical features of the AS patients**

| **Clinical features** | **Patients with AS** | |
| --- | --- | --- |
|  | N (total=302) ^#^ | **%** |
| Age (years±SD) | 39.52±10.91 |  |
| Male | 220 | 72.8 |
| Female | 82 | 27.2 |
| Radiologic sacroiliitis | 302 | 100 |
| Uveitis | 302 | 100 |
| HLA-B27(+) | 256 | 90.5 |
| HLA-B27(-) | 27 | 9.5 |

# In 302 AAU^+^AS^+^ patients, 283 were tested for HLA-B27, 256 (90.5%) were HLA-B27 positive, 27 (9.5%) were HLA-B27 negative and in 19 patients the HLA-B27 status was unknown.
