# Supplementary material for: A Functional Variant of PTPN22 Confers Risk for Vogt-Koyanagi-Harada Syndrome but Not for Ankylosing Spondylitis
Source: PLoS One. 2014 May 9;9(5):e96943. doi: 10.1371/journal.pone.0096943 (PMC4016172; doi:10.1371/journal.pone.0096943)
Supplement: Table S4 — Effects of PTPN22 SNPs on AAU+AS+ risk. (DOC) [file pone.0096943.s005.doc]

**Table S4. Effects of PTPN22 SNPs on AAU^+^AS^+^ r**isk

| **Genotype** | **AAU+AS+** | | **Controls** | | **P value** | **Pc value** | **OR (95%CI)** |
| --- | --- | --- | --- | --- | --- | --- | --- |
|  | N=302 | | N=2010 | |  |  |  |
| rs2488457 | N | % | N | % |  |  |  |
| GG | 41 | 13.6 | 310 | 15.4 | 0.404 | NS | 0.86(0.61-1.22) |
| CG | 141 | 46.7 | 1009 | 51.2 | 0.255 | NS | 0.87(0.68-1.11) |
| CC | 120 | 39.7 | 691 | 34.4 | 0.069 | NS | 1.26(0.98-1.61) |
| G Allele | 223 | 36.9 | 1629 | 40.5 | 0.092 | NS | 0.86(0.72-1.03) |
| C Allele | 381 | 63.1 | 2391 | 59.5 | 0.092 | NS | 1.16(0.98-1.39) |
| rs1310182 |  |  |  |  |  |  |  |
| CC | 17 | 5.6 | 84 | 4.2 | 0.250 | NS | 1.37(0.80-2.34) |
| CT | 95 | 31.5 | 589 | 29.3 | 0.445 | NS | 1.11(0.85-1.44) |
| TT | 190 | 62.9 | 1337 | 66.5 | 0.218 | NS | 0.85(0.66-1.10) |
| C Allele | 129 | 21.4 | 757 | 18.8 | 0.141 | NS | 1.17(0.95-1.44) |
| T Allele | 475 | 78.6 | 3263 | 81.2 | 0.141 | NS | 0.85(0.69-1.05) |
| rs3789604 |  |  |  |  |  |  |  |
| TT | 179 | 59.3 | 1254 | 62.4 | 0.298 | NS | 0.88(0.69-1.12) |
| GT | 104 | 34.4 | 651 | 32.4 | 0.479 | NS | 1.10(0.85-1.42) |
| GG | 19 | 6.3 | 105 | 5.2 | 0.443 | NS | 1.22(0.74-2.02) |
| T Allele | 462 | 76.5 | 3159 | 78.6 | 0.245 | NS | 0.89(0.72-1.09) |
| G Allele | 142 | 23.5 | 861 | 21.4 | 0.245 | NS | 1.13(0.92-1.38) |

P_c_=Bonferroni corrected P value. NS=Not significant. OR=odds ratio. 95% CI=95% confidence interval
